# The Effect of Alendronate on Proteome of Hepatocellular Carcinoma Cell Lines

**DOI:** 10.1155/2014/532953

**Published:** 2014-02-06

**Authors:** Amber Ilyas, Zehra Hashim, Nadia Naeem, Kanwal Haneef, Shamshad Zarina

**Affiliations:** ^1^National Center for Proteomics, University of Karachi, Karachi 75270, Pakistan; ^2^Dr. Panjwani Center for Molecular Medicine and Drug Research, ICCBS, University of Karachi, Karachi 75270, Pakistan

## Abstract

Cancer is a life threatening disorder effecting 11 million people worldwide annually. Among various types of cancers, Hepatocellular carcinoma (HCC) has a higher rate of mortality and is the fifth leading cause of cancer related deaths around the world. Many chemotherapeutic drugs have been used for the treatment of HCC with many side effects. These drugs are inhibitors of different cell regulatory pathways. Mevalonate (MVA) pathway is an important cellular cascade vital for cell growth. A variety of inhibitors of MVA pathway have been reported for their anticancerous activity. Bisphosphonates (BPs) are members of a family involved in the treatment of skeletal complications. In recent years, their anticancer potential has been highlighted. Current study focuses on exploring the effects of alendronate (ALN), a nitrogen containing BP, on hepatocellular carcinoma cell line using genomic and proteomics approach. Our results identified ten differentially expressed proteins, of which five were up regulated and five were down regulated in ALN treated cells. Furthermore, we also performed gene expression analysis in treated and control cell lines. The study may help in understanding the molecular mechanism involved in antitumor activity of ALN, identification of possible novel drug targets, and designing new therapeutic strategies for HCC.

## 1. Introduction

Hepatocellular carcinoma is the most common form of cancer worldwide and is third most frequent cause of cancer related death [1]. In 2008, approximately 696,000 deaths were reported all over the world from HCC. The incidence of HCC is high in Eastern and South-Eastern Asia [2]. It is also the most common cause of death among cirrhosis patients [3].

Various risk factors are involved in disease onset such as tobacco, aflatoxin B1, vinyl chloride, alcohol abuse, diabetes, obesity, nonalcoholic fatty liver disease, and hemochromatosis. Hepatitis B and C infection are the most common risk factors of HCC development [4]. The prognosis of HCC is poor as compared to high incidence of recurrence due to the late detection of the disease.

Treatment strategies for early stage HCC are well established including percutaneous ethanol injection, (PEIT) [5], microwave coagulation therapy (MC) [6], transcatheter arterial embolization [7], radiofrequency ablation (RFA) [8], and hepatic resection [5]. At advanced stages, however, all these treatment modalities fail and the only option left is of palliative chemotherapy. Palliative therapy does not completely cure the disease but can only improve the survival rate and the quality of life. Since existing chemotherapeutic drugs have many side effects, identification of an effective drug remains a hot research area.

Mevalonate pathway is an important target for anticancer drugs as it governs cell cycles including cell growth and survival. Manipulation of this pathway results in alteration of cancerous cell growth [9]. Mevalonate (MVA) is synthesized from the 3-hydroxy-3-methylglutaryl coenzyme A (HMG-Co A) [10]. Isoprenoids farnesyl pyrophosphate (FPP) and geranylgeranyl pyrophosphate (GPP), the end products of this pathway, play fundamental role in activation of intracellular Ras and Ras related GTP binding proteins which are critical for cell signaling cascade [11]. Approximately in 20% of all human tumors, inactivation of GTP hydrolysis occurs due to mutated Ras protein [12, 13] resulting in permanent activation of cell cycle, uncontrolled growth, and cell proliferation [14].

Bisphosphonates (BPs) are pyrophosphate analogues and are inhibitors of mevalonate (MVA) pathway, mainly Farnesyl pyrophosphate synthase (FDPS), thus preventing prenylation of small signaling proteins (Ras, Rho, and Rab) [15, 16]. BPs are potential inhibitors of bone resorption [17] and during myeloma and metastatic phase of breast and prostate cancer, BPs are used to cure the skeletal complications [18]. Nitrogen containing bisphosphonates (N-BPs) have been used for the treatment of osteoporosis and tumor related hypercalcemia [19]. There has been increasing evidence that BPs can inhibit proliferation and induce apoptosis in a variety of human tumor cells like myeloma, breast, pancreas, and prostate under various conditions [20–22]. *In vitro* studies have indicated that BPs can cause induction of apoptosis in human myeloma cell lines [23]. Alendronate (ALN), a BPs family member, has shown to inhibit invasion of prostate cancer [24], proliferation/invasion in human epidermal carcinoma cells [25] and to stimulate inhibitors of DNA binding/differentiation genes in C2C12 cells [26]. These studies mainly refer to cell cycle arrest and apoptotic pathway induction and very few studies have been conducted at proteomics level.

Till date, no work has been reported on proteomic profiling on effects of ALN in HCC cell lines. The objective of the current study was to evaluate the effects of ALN on HCC cell lines and examine altered gene and protein expression in response to drug treatment. We have performed proteome profiling of hepatocellular carcinoma Huh-7 cells treated with ALN and control. Furthermore, we have observed the gene expression pattern of farnesyl pyrophosphate synthase (*FDPS*), farnesyl-diphosphate farnesyl transferase 1 (*FDFT1*), caspase 7 (*CASP7*), Rho family GTPase (*RND3*), Ras oncogene family member (*RAB11A*), and DNA methyl transferase 1 (*DNMT1*) in ALN treated and control Huh-7 cells.

## 2. Materials and Method

### 2.1. Cell Culture and Treatment

Human hepatocellular carcinoma cell line Huh-7 were grown in Dulbecco's Modified Eagle's Medium (DMEM) with 4.5 g/L glucose, supplemented with 10% fetal bovine serum (FBS), 2 mM glutamine, 100 U/mL penicillin and 100 *μ*g/mL streptomycin. Cells were maintained in humidified atmosphere of 5% CO_2_ at 37°C and routinely sub cultured using 0.05% trypsin-EDTA. The medium were changed after every two days. Exponentially growing cells were incubated with different concentrations of ALN (5, 10, and 20 *μ*M) for 24, 48, and 72 hrs.

### 2.2. Cytotoxicity Assay

Approximately 1.2 × 10^6^ cells/mL (1000 *μ*L/well) were seeded in 6-well plates (Corning). Cells were incubated for 24 hrs at 37°C in 5% CO_2_ to allow cell attachment before performing cytotoxicity assay. After 24 hrs incubation, fresh medium containing different concentrations of the ALN (5 *μ*M, 10 *μ*M, and 20 *μ*M) were added to the wells and incubated for 24, 48, and 72 hrs. After each respective time interval, media were taken and cytotoxicity assay was performed using CytoTox 96 Nonradioactive Cytotoxicity Assay (Promega) according to manufacturer's instructions. Absorbance was taken at 490 nm using microplate reader (Backmann Coulter). Cell cytotoxicity was expressed as percentage against the control wells. All assays were performed in triplicate.

### 2.3. Analysis of Cell Cycle Using Flow Cytometry

Cells were cultured with 5 *μ*M ALN for 24, 48, and 72 hrs. After respective time interval, cells were harvested by trypsinization and washed twice with PBS. Cells were fixed in ice cold 70% ethanol, washed, and resuspended in 1 mL PBS. Cells were treated with 10 *μ*L (100 units/mL) RNase for 30 mins in water bath (37°C) and stained with 150 *μ*L (20 *μ*g/mL) propidium iodide (PI) for 30 mins at room temperature. Stained cells were then analyzed by flow cytometer (FACSCalibur, Becton Dickinson, USA) and DNA content was quantified by using Flow Jo software. Samples were analyzed in duplicate.

### 2.4. Reverse Transcriptase-PCR

Total RNA was extracted from treated and untreated (control) cells using SV Total RNA extraction kit (Promega) according to manufacturer's protocol. Purity of the extracted RNA was measured by 260/280 ratio. ~4 *μ*g RNA was reverse transcribed using superscript RT kit (Invitrogen). PCR was performed to amplify the transcript of the Human *FDPS*, *FDFT1*, *CASP7, RAB11A, RND3,* and *DNMT1* genes using oligonucleotide primers. Human **β*-Actin* gene was used as an internal standard. PCR conditions were: denaturation at 94°C for 1 min, annealing at 60–62°C for 1 min, and extension at 74°C for 1 min. Total number of amplification cycles were 30. The primer sequences, expected product size, and annealing temperatures are listed in Table 1. PCR products were electrophoretically resolved on 2% agarose gel. Untreated Huh-7 cells and PCR blank were used as positive and negative control, respectively. Densitometry was performed to compare band densities using Quantity One Software (BioRad). The integrated density of each gene was normalized with the corresponding **β*-Actin* band density.

### 2.5. 2-DE Analysis

For the extraction of protein, 5 *μ*M ALN treated and untreated (control) cells were lysed in lysis buffer containing 8 M urea, 0.2 M EDTA, 0.5 M DTT, glycerol, NP-40, ampholyte solution pH 3–10 in 0.5 M Tris-HCl with protease inhibitor cocktail. The lysate was centrifuged at 12,000 ×g for 10 mins at 4°C. Protein concentrations were measured using Bradford protein estimation kit (BioRad). Approximately 80 *μ*g protein from the treated and untreated samples were dissolved in rehydration buffer containing 8 M Urea, 0.5% ampholyte solution, 0.2% DTT, 0.5% CHAPS, and few crystals of Bromophenol blue and used to rehydrate the IPG strip 3–10 NL over night at room temperature. IEF was performed on Multiphor II (GE Healthcare) system at 20°C using total of 7000 V/h. IPG Strips were equilibrated for 30 mins each using equilibration buffer 1 containing 0.5 M Tris-HCl (pH 6.8), 6 M urea, 2% SDS, 30% glycerol, and 2% DTT, then in equilibration buffer 2 consisting of 0.5 M Tris-HCl (pH 6.8), 6 M urea, 2% SDS, 30% glycerol, and 2.5% w/v iodoacetamide. After equilibration, strips were applied on 12% polyacrylamide gel to run second dimension SDS-PAGE at a constant voltage of 65 volts using mini protean electrophoresis system (BioRad). Gels were stained overnight using coomassie brilliant blue G-250.

### 2.6. Image Analysis

Gel images were acquired using ExQuest spot cutter and analyzed by PDQuest software (BioRad). For the differential protein expression analysis, a master gel image was prepared from control cells showing highest number of spots with best resolution. Spots from 5 *μ*M ALN treated gel were matched to the master gel and spot density was determined.

### 2.7. Protein Identification Using LC-MS/MS

To identify expressed proteins, gel spots were excised and digested with trypsin. Tryptic peptides were subjected to nLC-MS/MS analysis using Thermo LTQ XL linear ion trap MS interfaced with nano LC system. 1 *μ*L sample was injected through an autosampler into the nLC system at the flow rate of 300 nL/min. The peptides were eluted onto a 75 *μ*m I.D ×15 cm Pep-Map 100 C-18 nanocolumn. The column was equilibrated with 96.8% A (0.1% F.A) and 3.2% B (98% ACN, 2% water, 0.1% F.A). Peptide separation was achieved with multi-step gradient from 3.2% to 80% solution B over 70 mins. Silica tip 10 +/− 1 *μ*m emitter was used to apply the peptides on to LTQ MS through nanoelectrospray (NSI). The capillary voltage was set at 29.95 V and the capillary temperature was maintained at 270°C. Xcalibur software version 2.0.7SP1 was used to produce RAW files and Proteome Discoverer 1.2 to convert RAW files into MGF files (see Supplementary Material available online at http://dx.doi.org/10.1155/2014/532953). Protein identification was performed using Mascot search engine against latest version of Swiss-PROT. For Mascot search, following parameters were used: peptide mass tolerance ± 1.5 Da, MS/MS tolerance ± 0.5 Da, carbamidomethylation of Cys, and Met Oxidation as fixed and variable modifications, respectively.

### 2.8. Statistical Analysis

Statistical analyses were performed by SPSS 20 software. The paired *t*-test was used to compare the changes in gene expression and FACS results. Statistical analysis was done to evaluate the significant differences for cells proliferation, gene expression and FACS in ALN treated and untreated cells. The differences was statistically significant if *P* < 0.05.

## 3. Results

### 3.1. Effect of Alendronate on the Growth of Hepatocellular Carcinoma Cells *In Vitro*

**Figure 1:**
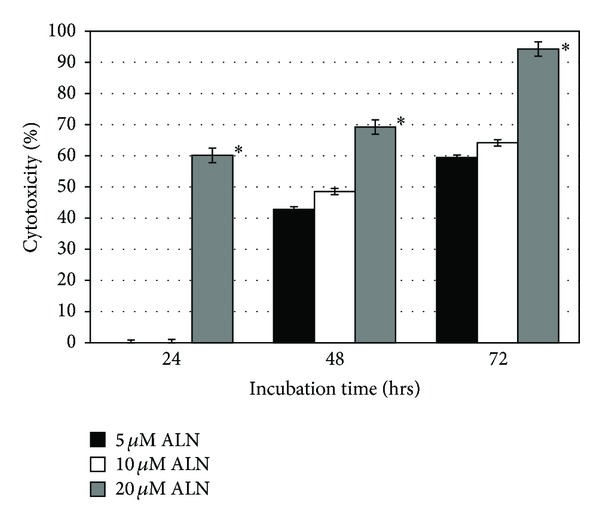
The effect of 5 *μ*M, 10 *μ*M, and 20 *μ*M ALN on Huh-7 cells after 24 hrs, 48 hrs, and 72 hrs treatment. Cell apoptosis was determined by cytotoxicity assay. Each bar represents mean ± S.D for 5 *μ*M, 10 *μ*M, and 20 *μ*M, respectively. * represents statistically significant value with reference to respective control (*P* < 0.05).

ALN inhibited the proliferation of HCC cell lines in dose and time dependent manner (Figure 1). The effect of ALN was studied at concentration ranging from 5 *μ*M to 20 *μ*M on Huh-7 cell viability. ALN induced concentration dependent reduction in cell viability (Figure 1). Cell death was found to be negligible after 24 hrs incubation at all concentrations except 20 *μ*M. After 48 hrs incubation at 5 *μ*M ALN, 42% inhibition in cell viability was observed which reached up to 60% after 72 hrs. Same trend was observed in case of 10 *μ*M concentration where 48 and 64% cytotoxicity was observed after 48 and 72 hrs, respectively. Maximum inhibition (94%) was observed at 72 hrs with 20 *μ*M concentration. Our results indicated 5 *μ*M concentration to be the most effective hence it was used for further studies.

### 3.2. ALN Induced Cell Cycle Alterations

**Figure 2:**
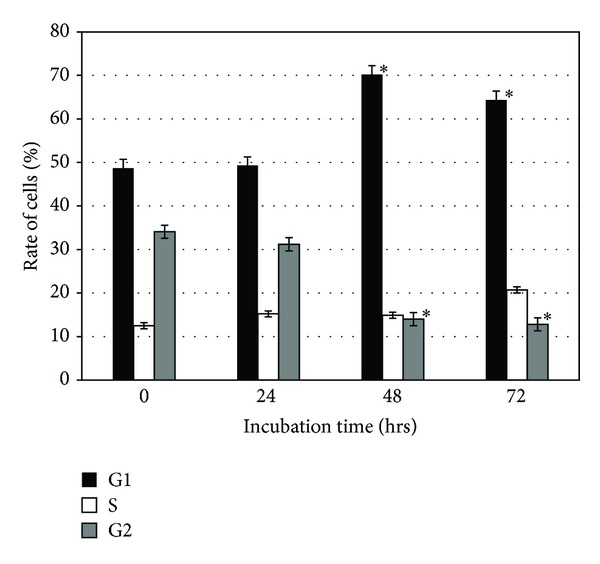
Quantification of the cell progression phases of ALN treated cells at 5 *μ*M for 24, 48, and 72 hrs by FACS. * represents statistically significant value with reference to respective control (*P* < 0.05).

Cell cycle analysis on ALN treated cells was done by Fluorescence activated cell sorting (FACS). Cell proliferation was inhibited by ALN in Huh-7 cells. Cells treated with 5 *μ*M ALN for 24 hrs showed no change in cell proliferation while 48 hrs and 72 hrs ALN treatment showed increase cell proliferation at G1 and decrease at G2 phase (Figure 2).

### 3.3. Differential Gene Expression Analysis of the Human Hepatocellular Carcinoma Huh-7 Cells

**Figure 3:**
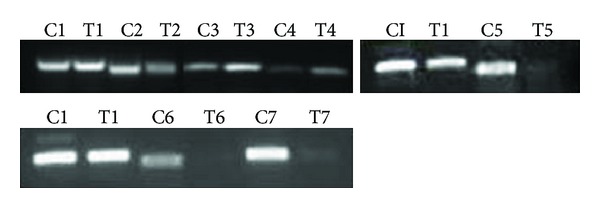
Expression level of **β*-Actin* (C1, T1), *FDPS* (C2, T2), *FDFT1* (C3, T3)*, CASP7* (C4, T4), *DNMT1* (C5, T5)*, RAB11A* (C6, T6), and *RND3* (C7, T7) mRNA in 5 *μ*M ALN treated (T) and untreated (C) Huh 7 cells.

**Figure 4:**
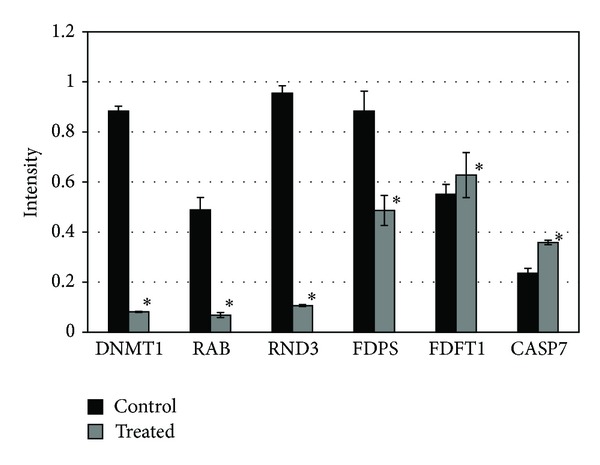
The corresponding expression abundance of *DNMT1*, *RAB11A*, *RND3*, *FDPS, FDFT1,* and *CASP7* mRNA in 5 *μ*M ALN treated and control Huh 7 cells. * represents statistically significant value (*P* < 0.05).

Reverse transcription of mRNA extracted from ALN treated and untreated cells showed *FDPS, FDFT1, CASP7, RAB11A, RND3,* and *DNMT1* gene expression (Figures 3 and 4). ALN treated cells showed less expression of the *FDPS* gene as compared to control whereas the expression of *FDFT1* and *CASP7* gene was found to be increased. Expression levels of *RAB11A, RND3,* and *DNMT1* were also decreased in treated cells as compared to untreated cells. **β*-Actin* expression was same in treated and untreated Huh-7 cells. Results are represented as mean ± S.D while *P* < 0.05 was used as statistically significant difference.

### 3.4. Mass Spectrometry Identification of Differentially Expressed Proteins

**Figure 5:**
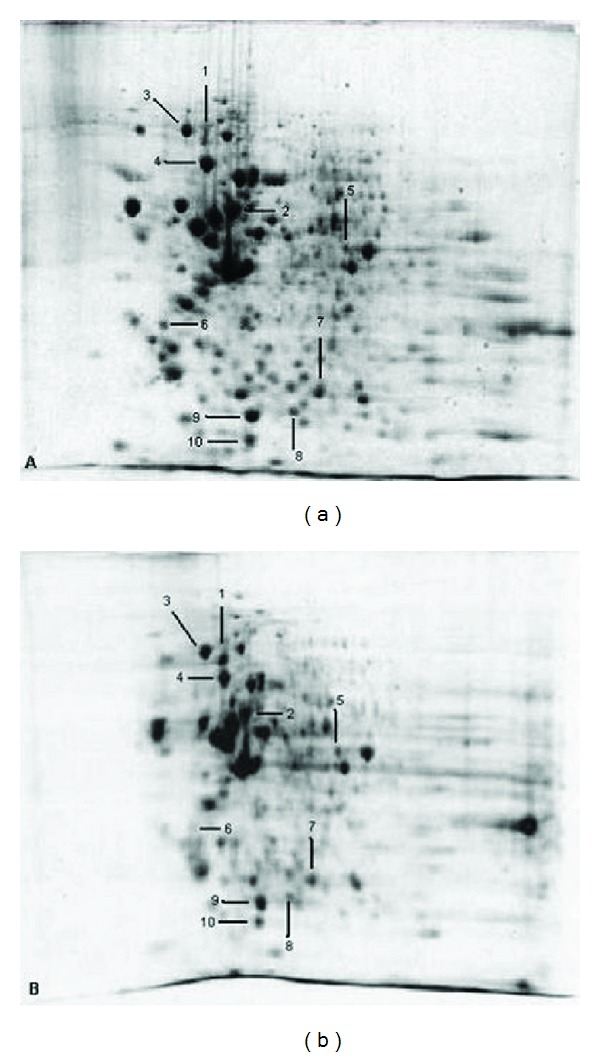
Differential proteomic analysis of 5 *μ*M ALN treated (b) and untreated control (a) Huh-7 cells using 2D gels. Differentially expressed spots identified by MS/MS analysis are marked with arrows.

**Figure 6:**
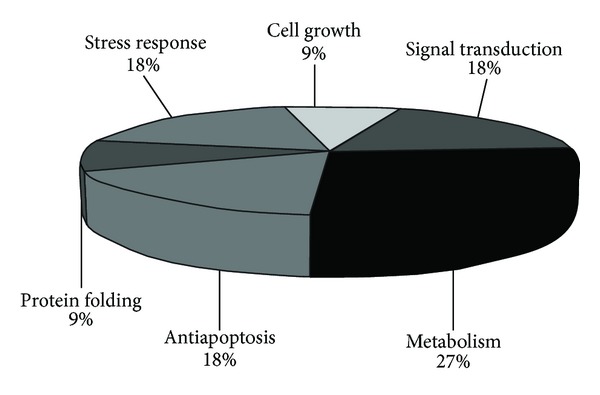
Distribution of identified proteins according to cellular function.

Figures 5(a) and 5(b) show two dimensional gel electrophoresis patterns of untreated and ALN treated HCC cell lines, respectively. Ten differentially expressed spots were identified by LC-MS/MS analysis as shown in Table 2. Among these, 5 proteins were found to be upregulated, whereas 5 were downregulated in ALN treated HCC cell lines. The identified proteins were classified according to their biological function in order to evaluate their association with HCC and ALN treatment and were found to be mainly involved in cellular growth, signal transduction pathways, and metabolism (Figure 6).

## 4. Discussion

BPs are synthetic analogues of drugs commonly used for the treatment of osteoporosis and in cancer-induced bone diseases [19]. There has been an increasing evidence that they have anticancerous activity and can induce tumor cell death through induction of apoptosis. BPs have shown promising inhibitory effect on tumor cell proliferation/survival or on cell invasiveness in human epidermoid carcinoma, breast cancer, and small cell lung carcinoma [25, 27, 28].

Earlier incubation studies on cancer cell lines indicated that N-BPs are effective in high concentrations [29]. N-BPs such as pamidronate, zoledronate, or ibandronate were used in 100 *μ*mol/L concentrations to induce apoptosis in breast and prostate cancer cells. ALN, on the other hand, has shown to inhibit invasion of prostate cancer *in vitro* at low concentration [24]. It also prevents migration of the cells without affecting the apoptosis *in vitro* [30]. These reports have suggested that ALN might be effective in low and moderate concentrations *in vivo*. In present study, ALN has shown direct cytotoxic effect on hepatocellular carcinoma cell lines. We observed 5 *μ*M to be an effective concentration that induced 42% cell death after 48 hrs incubation hence substantiating earlier reports. We also found significant antiproliferative effects of ALN on HCC. Our findings are in agreement with previous report suggesting ALN is effective in time and dose dependent manner [31].

**Figure 7:**
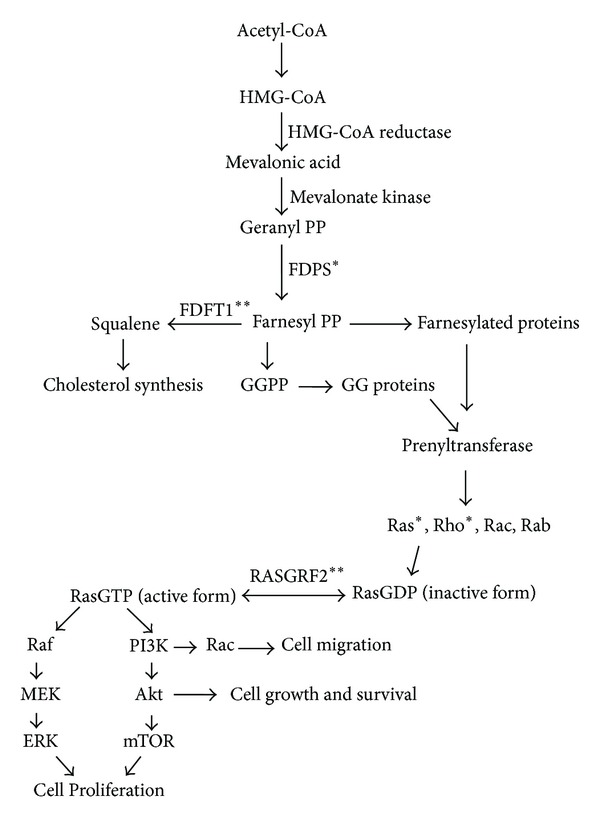
Flow diagram indicating mevalonate pathway. PP (pyrophosphate), FDPS (Farnesyl pyrophosphate synthase), FDFT1 (farnesyl-diphosphate farnesyltransferase 1), FPP (Farnesylpyrophosphate), GGPP (geranylgeranyl pyrophosphate), Rho, Ras, Rab, Rac (small GTPases), and RASGRF2 (Ras-specific guanine nucleotide-releasing factor). ** and * show up and down regulation of genes and proteins respectively as observed in current study.

N-BPs are potent inhibitors of MVA pathway as postulated in Figure 7 [32]. The molecular mechanism they work through involves binding of N-BPs with key regulatory enzyme, FDPS, resulting in inhibition of prenylation of small signaling proteins including Rho, Ras, and Rab. Treatment of Huh-7 cells with ALN resulted in apoptosis through inhibition of *FDPS*. We observed decreased mRNA expression of *FDPS* in ALN treated cells confirming that the mode of action of ALN is through regulating *FDPS*. Effect of ALN on FPP and GGPP can be rescued by the addition of FPP and GGPP that ultimately save the activity of Ras and Rho family proteins [33]. The branching point in MVA pathway involves FPP, the product of FDPS, which serves as a precursor for cholesterol synthesis and protein prenylation [34]. A recent study has reported that N-BPs mediate their effect through suppression of GGPP synthetic pathway [35] while cholesterol synthesis pathway remains unaffected [36]. To examine the effect of ALN on protein prenylation, expression of *RAB11A* and *RND3* genes (corresponding to Rho and Ras protein families resp.) was studied which was found to be decreased. We also checked expression of *FDFT1* (gene encoding squalene synthase, first enzyme of cholesterol synthesis) and found it to be up regulated in ALN treated cells. Indeed, squalene synthase is not a possible target of ALN inhibition [37] and ALN is proposed to be a poor inhibitor in contrast to other N-BPs such as incadronate and ibandronate [38]. BPs are reported to act through changes in CpG-methylation state of gene promoter regions involved in the cell proliferation and apoptosis [39]. To examine the effect of ALN on promoter methylation, we examined gene expression of *DNMT1*, an enzyme responsible for CpG methylation during cell replication [40]. In ALN treated cells, decreased expression of *DNMT1* was observed suggesting BPs can modulate CpG methylation of gene promoters.

Caspases (cysteinyl aspartate-specific protease) are the key regulatory enzymes of the apoptotic cascades [41]. The gene expression of *CASP7* was analyzed to investigate the molecular mechanism of apoptosis induced by ALN. Earlier studies showed that the expression of cleaved *caspases 6*, *7*, and *9* is increased in time and concentration dependent manner during apoptosis [42]. We also found the increased expression of *CASP7* gene in ALN treated Huh 7 cells. Our results prove that in ALN treated cells, apoptotic pathway is progressed due to which increased *CASP7* gene expression was observed.

To compliment gene expression study, we analyzed differential proteome profile of ALN treated and control cells. We have performed first proteomics study of hepatocellular carcinoma Huh-7 cell lines treated with ALN. We identified 5 up regulated and 5 down regulated proteins with different biological functions (Table 2). Most of the identified proteins have been reported to be involved in HCC progression, transcription, and cell growth (Figure 6).

In our study, elevated expression of peroxiredoxin 2 (Prx 2) was observed in ALN treated cells. Prx 2 is a member of peroxiredoxin protein family that provides cellular defense against oxidative stress [43]. Prx 2 is also involved in cell signaling pathways of tumor necrosis factor-*α* and growth factor by regulating intracellular levels of H_2_O_2_ [44, 45]. A recent study reported low expression of Prx 2 in human melanoma [46] which is most likely due to hyper-methylation of CpG island in promoter region [47]. In HCC tissue, Prx 2 was down regulated hence was considered as a tumor suppressor [48]. Our *in vitro* study revealed an up-regulation of Prx 2 in ALN treated cells substantiating its role as a possible tumor suppressor. This result is also supported by decreased expression of *DNMT1* as observed in this study.

Ras protein-specific guanine nucleotide-releasing factor 2 (RASGRF2) is a Ras signaling protein [49] which regulates conversion of active/inactive forms of Ras proteins. Our results show a decreased expression of RASGRF2 in ALN treated cells. Indeed, this observation substantiates results of gene expression analysis and suggests the pathway of ALN leading to tumor cell death or apoptosis.

We report decreased expression of IL-7 and STAT protein in ALN treated Huh-7 cells. Interleukin (IL) 7 is a cytokine that stimulates the development and proliferation of malignant cells such as in leukaemia and lymphoma [50]. IL-7 gene is expressed in many solid tumors including head and neck squamous cell carcinoma [51], renal [52], esophageal [53], and Warthin's tumour of the parotid gland [54]. IL-7 binding to its ligand triggers a series of intracellular phosphorylation events such as activation of phosphoinositide 3 kinase (PI3-K), Janus kinases (JAK-1 and JAK-3), signal transducers and activators of transcription (STAT) [55]. These molecules are involved in many cellular processes such as cell adhesion, cellular differentiation, motility, and mitogenesis [56, 57].

## 5. Conclusion

Current *in vitro* study presents first report on proteomics analysis of ALN treated hepatocarcinoma cell line Huh-7. Our study provides a baseline proteomics data and confirms that the mode of action of ALN is through mevalonate pathway. Furthermore, ALN in moderate concentration is sufficient to activate apoptotic pathway and may provide a strategic platform to develop novel compounds for chemotherapies of human malignancies.

## Supplementary Material

Screen shots of RAW files of all identified proteins generated by Thermo LTQ XL linear trap Mass spectrometer. These MS/MS aggregated scans show the relative abundance of peptides in particular protein sample. The RAW files are converted into MGF files using Proteome Discoverer 2.0 software.

## Figures and Tables

**Table 1:** Forward and reverse primer sequences, product size, and annealing temperatures used in the study.

S. no.	Gene	Primer sequence	Tm	Product Size
1	*β-Actin *	F: 5′GGACTTCGAGCAAGAGATGG	62.4°C	234 bp
		R: 3′AGCACTGTGTTGGCGTACAG		

2	*FDPS *	F: 5′AGGGCAATGTGGATCTTGTC	60.4°C	180 bp
		R: 3′GAAAGAACTCCCCCATCTCC		

3	*FDFT1 *	F: 5′GGTCCCGCTGTTACACAACT	60.4°C	194 bp
		R: 3′AAAACTCTGCCATCCCAATG		

4	*CASP7 *	F: 5′AGTGACAGGTATGGGCGTTC	61.4°C	164 bp
		R: 3′CGGCATTTGTATGGTCCTCT		

5	*DNMT1 *	F: 5′GTGGGGGACTGTGTCTCTGT	62.4°C	204 bp
		R: 3′TGAAAGCTGCATGTCCTCAC		

6	*RAB11A *	F: 5′CCACCACGACTGGCTAATTT	60°C	208 bp
		R: 3′ATCAAGGCACCATGGCTAAC		

7	*RND3 *	F: 5′GTGCTTGCATTTTTGGGTTT	60°C	226 bp
		R:3′ATCCCATGGGTCCTGATACA		

**Table 2:** Differentially expressed proteins identified with nano LC-MS/MS from Huh-7 cell lines.

Spot no.	Protein Name	Accession no.	Sequence coverage%	Mol·wt·kDa	pI	Protein expression	Peptide matches	Function
1	Heat shock protein 90	P08238	6	90	4.73	↑	3 (1)	Stress responce
2	60 kDa Heat shock protein	P10809	34	61	5.7	↑	28 (17)	Protein folding
3	Protein dicaudal D homolog 1 isoform 1	Q96G01	6	111	5.6	↓	9 (3)	Interacts with RAB6A
4	Signal transducer and activator of transcription 1-alpha/beta	P42224	4	87	5.7	↓	3 (1)	Signal transduction
5	Ras-specific guanine nucleotide-releasing factor	Q86X27	3	65	8.86	↓	3 (2)	Signal transduction
6	Annexin A5	P08758	24	36	4.9	↑	8 (6)	Blood coagulation, haemostasis, signal transduction
7	Triosephosphate isomerase	P60174	13	26.8	7.10	↑	2 (2)	Metabolism
8	Interleukin-7	P13232	8	20	8.87	↓	5 (1)	Growth factor activity
9	Glutathione S-transferase P	P09211	49	235	5.4	↓	13 (11)	Antiapoptosis, detoxification
10	Peroxiredoxin-2	P32119	29	22	5.66	↑	5 (5)	Antiapoptosis, signal transduction
