# Supplementary figures and images for: The Effect of Alendronate on Proteome of Hepatocellular Carcinoma Cell Lines
Source: Int J Proteomics. 2014 Feb 6;2014:532953. doi: 10.1155/2014/532953 (PMC3932719; doi:10.1155/2014/532953)

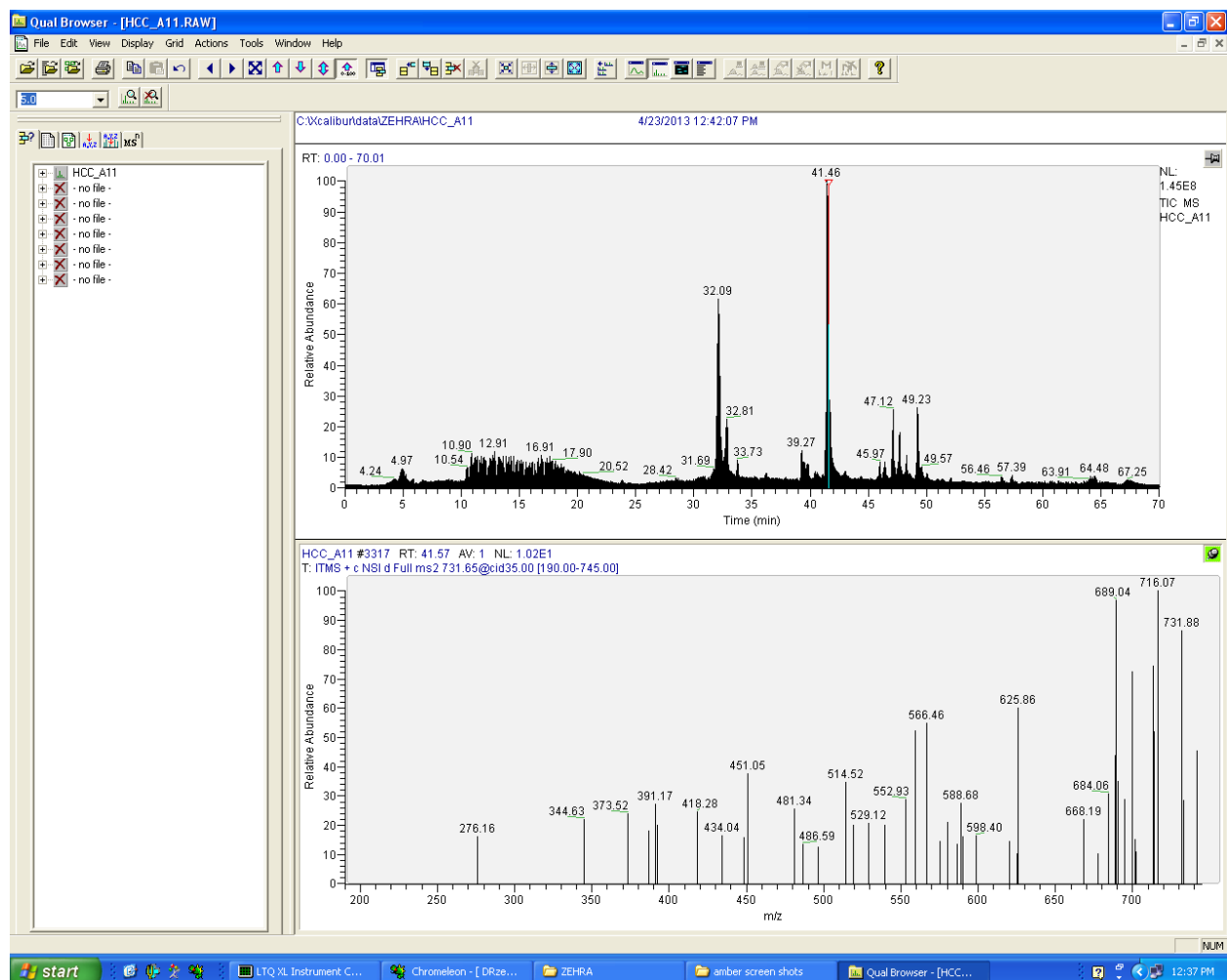

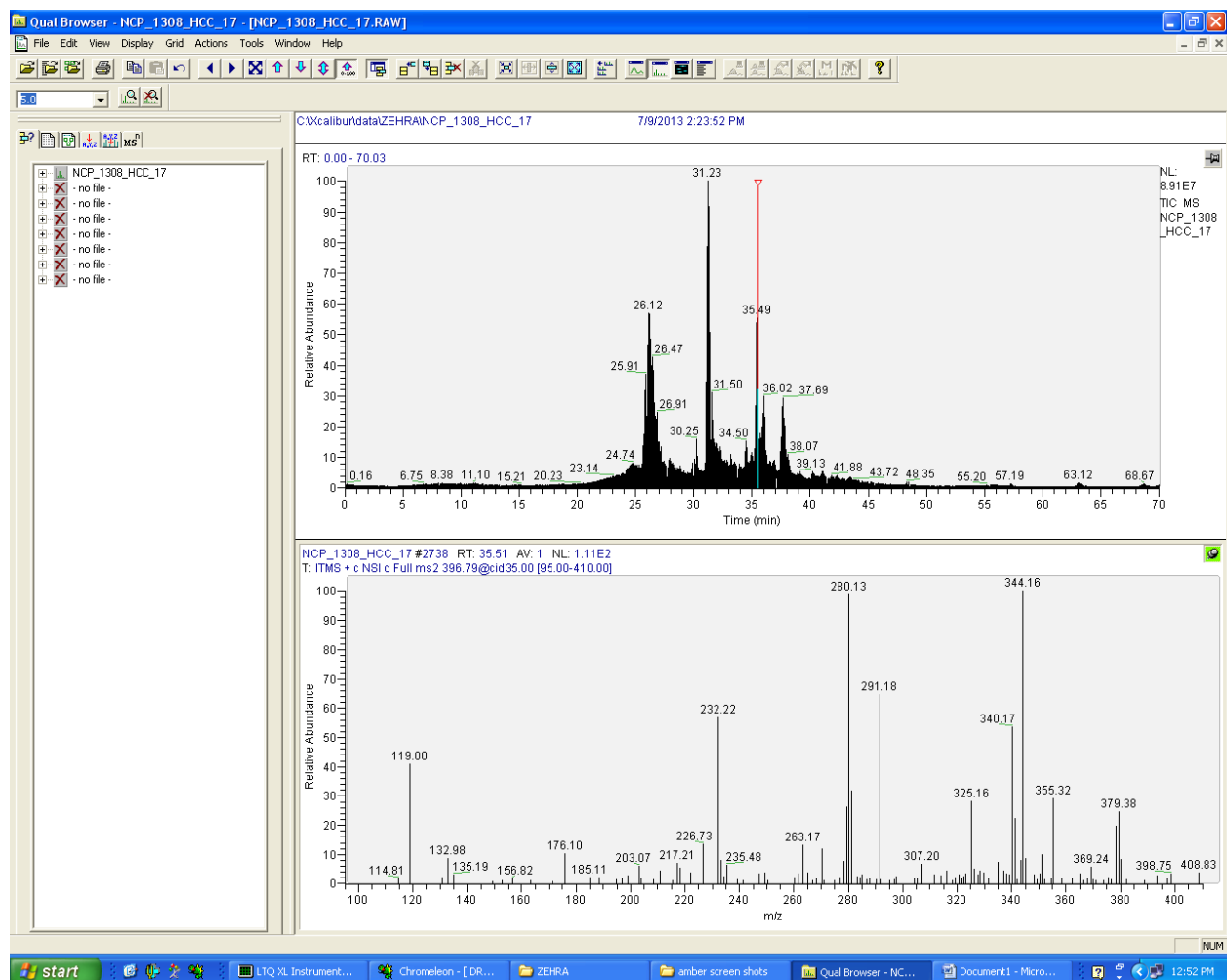

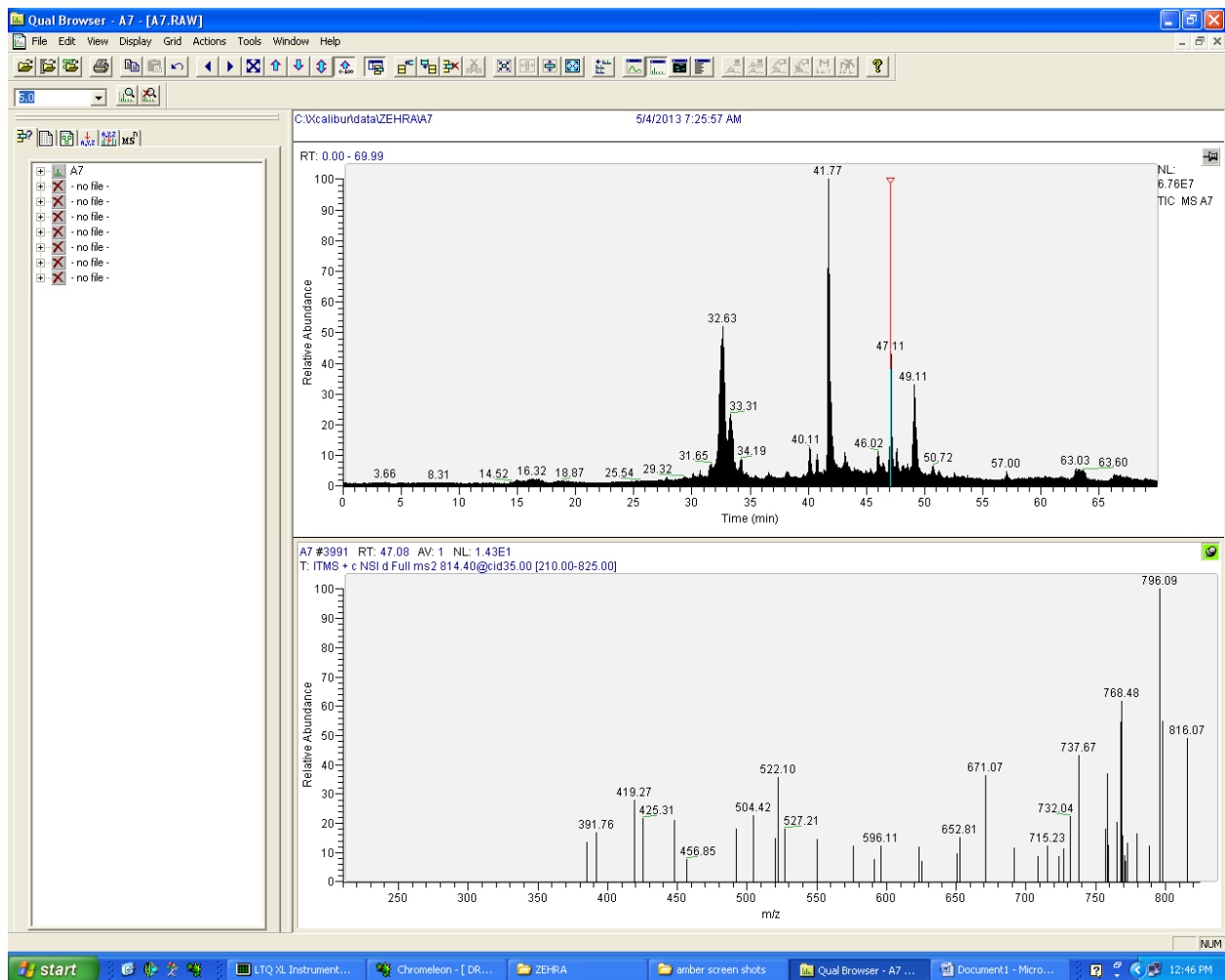

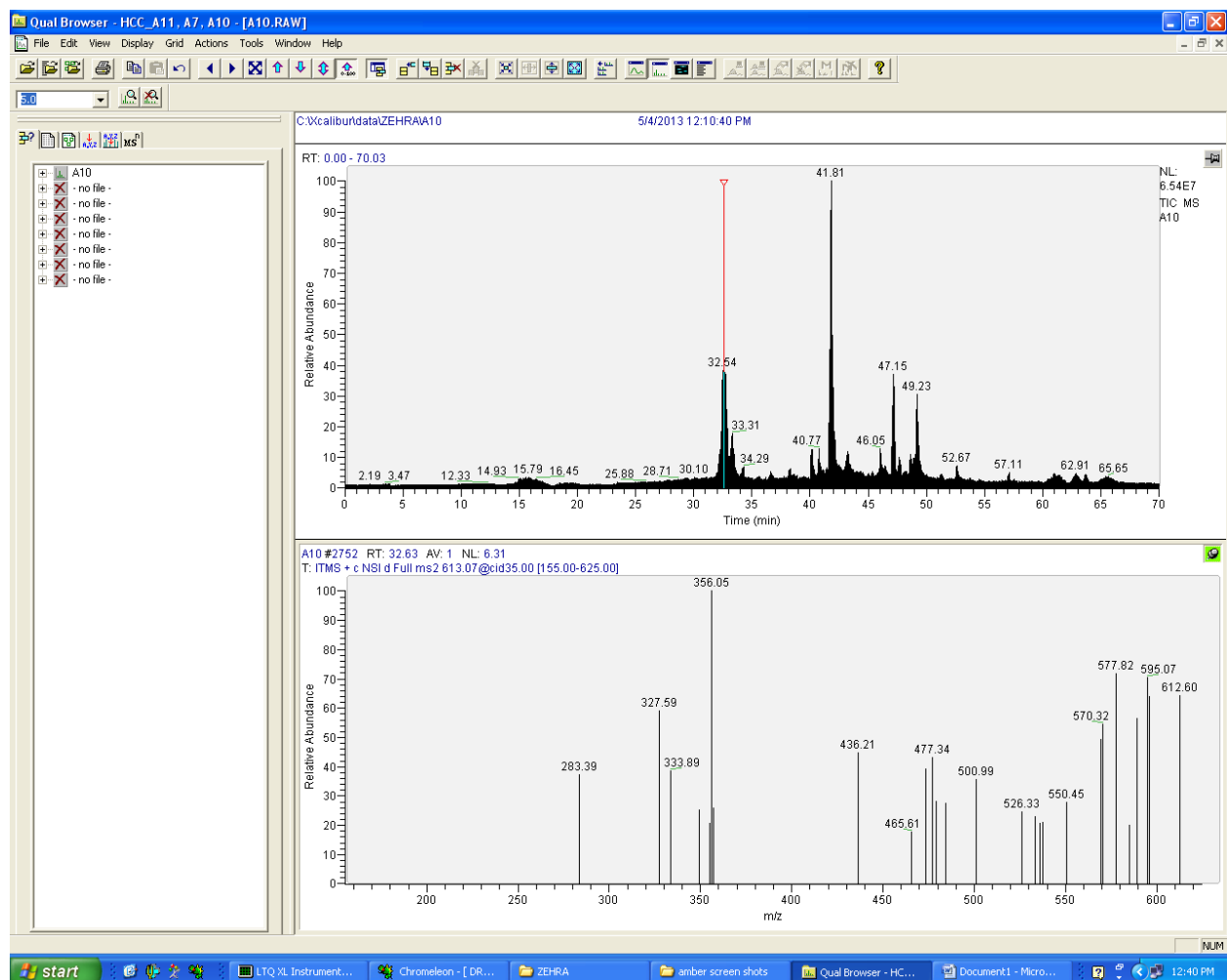

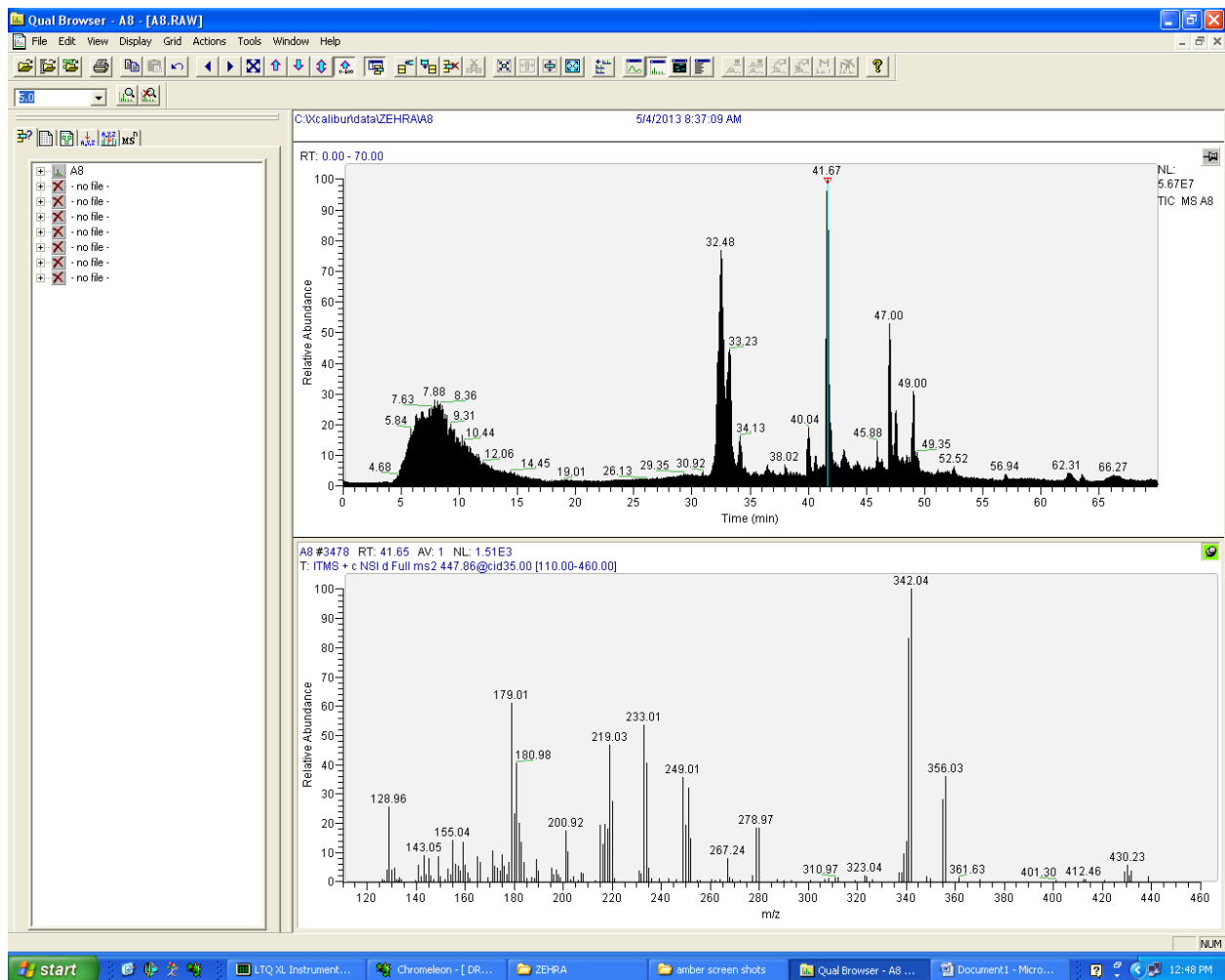

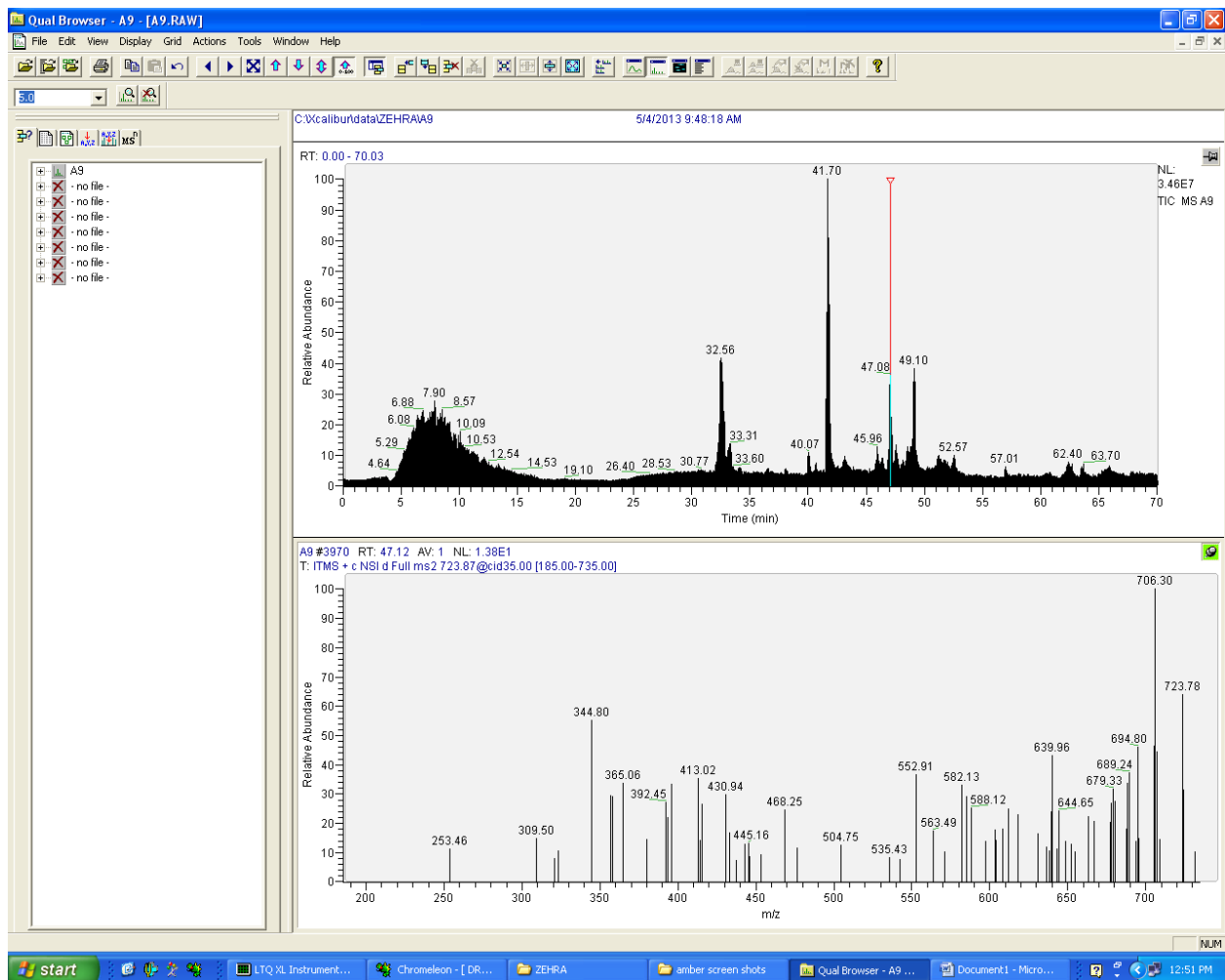

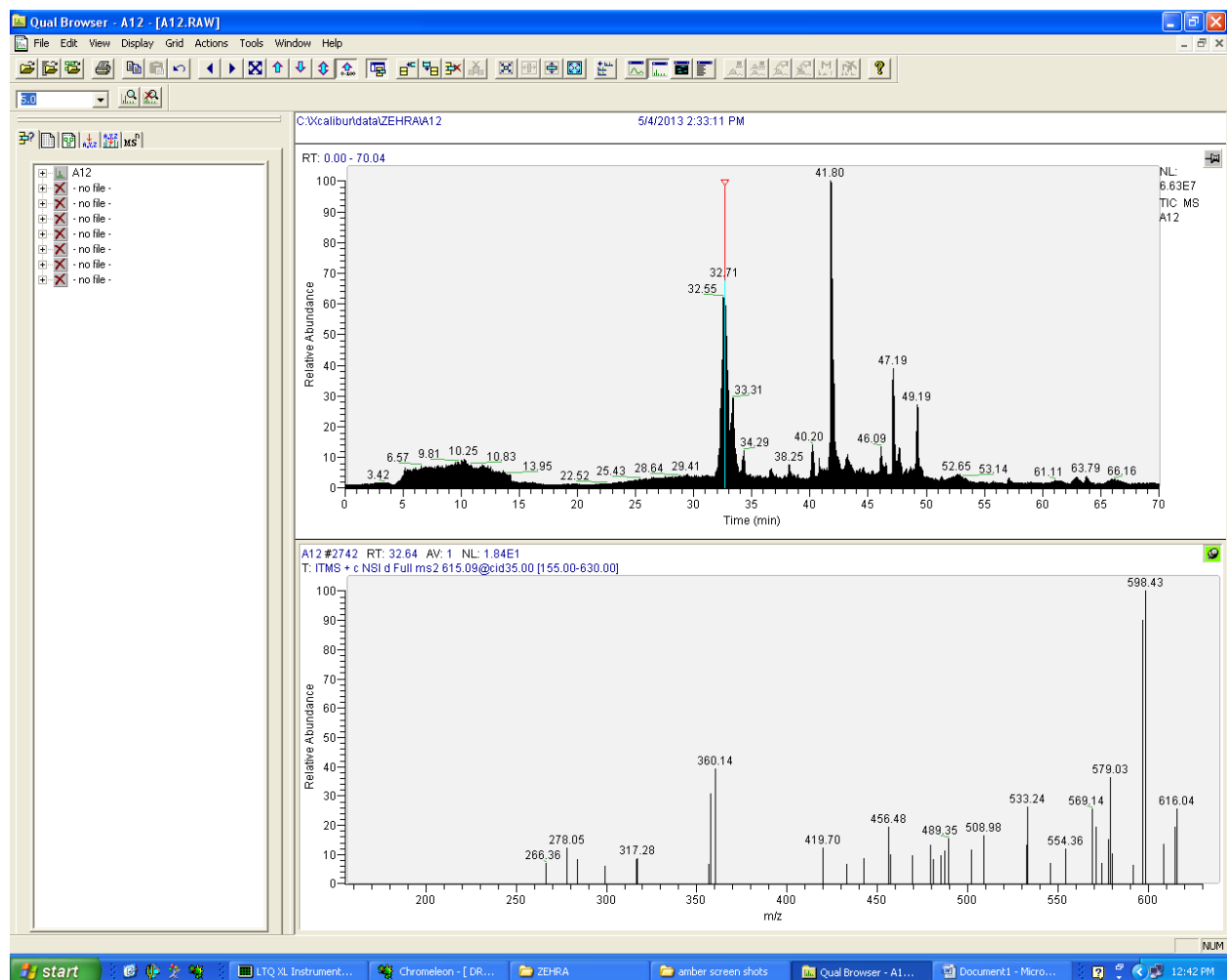

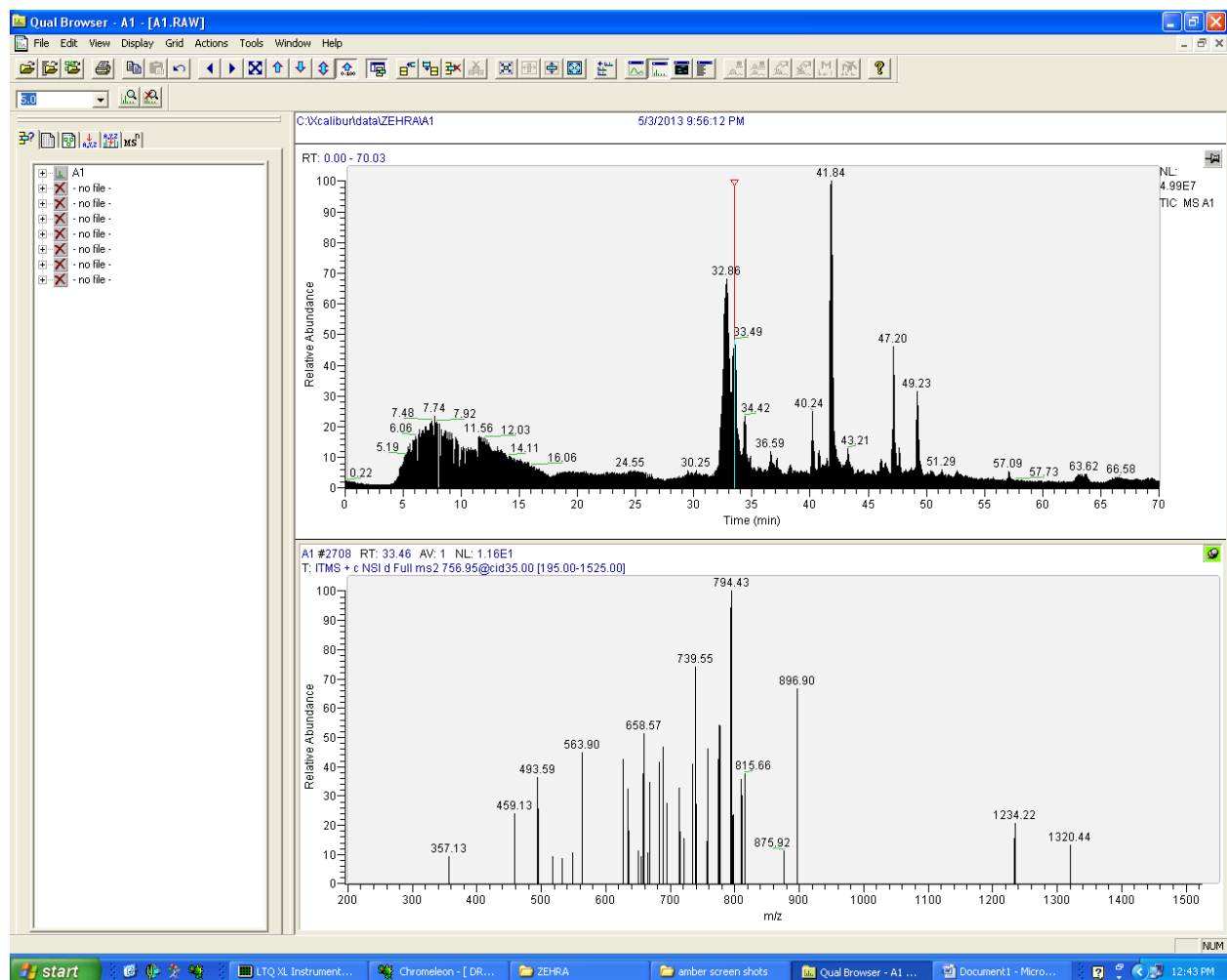

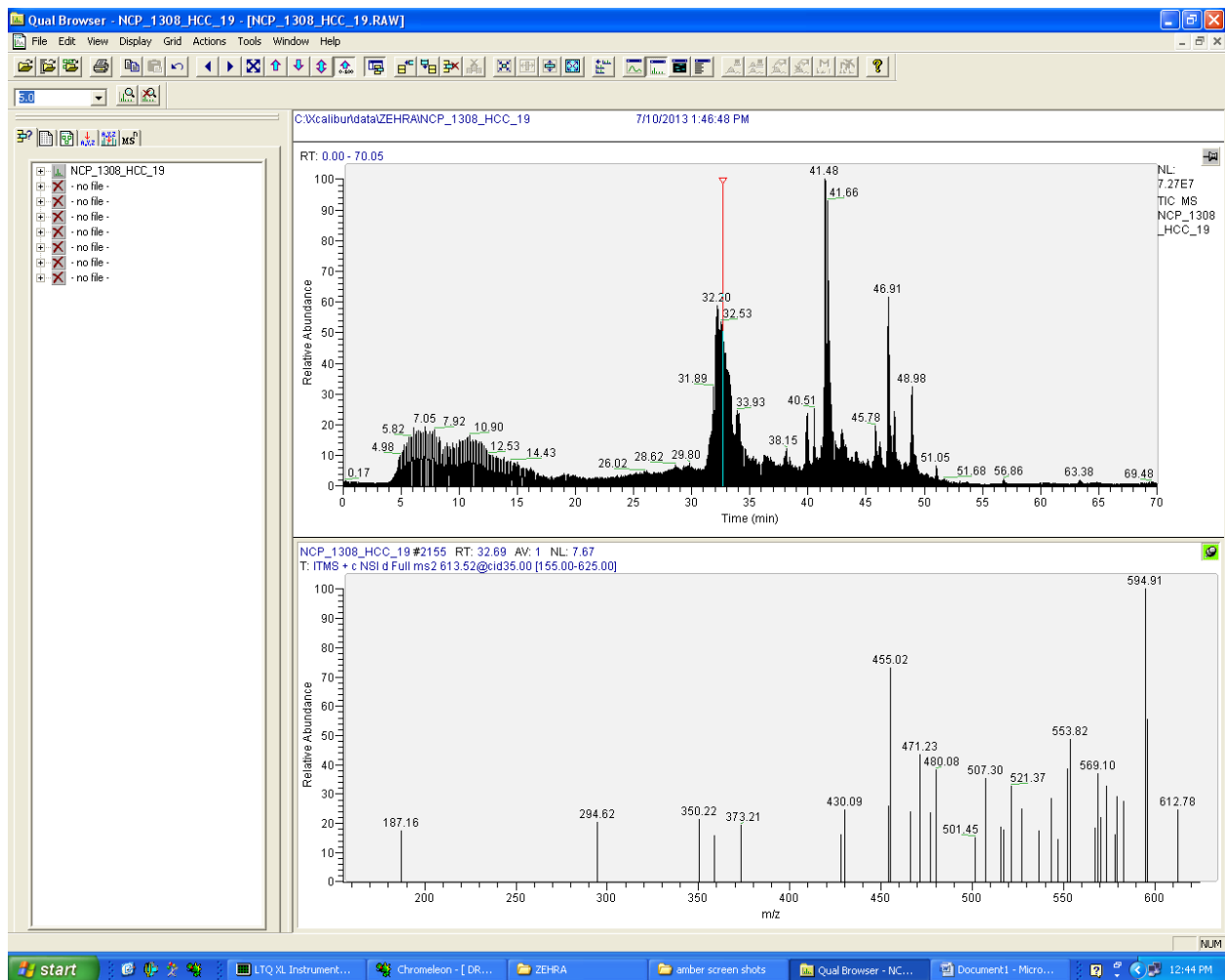

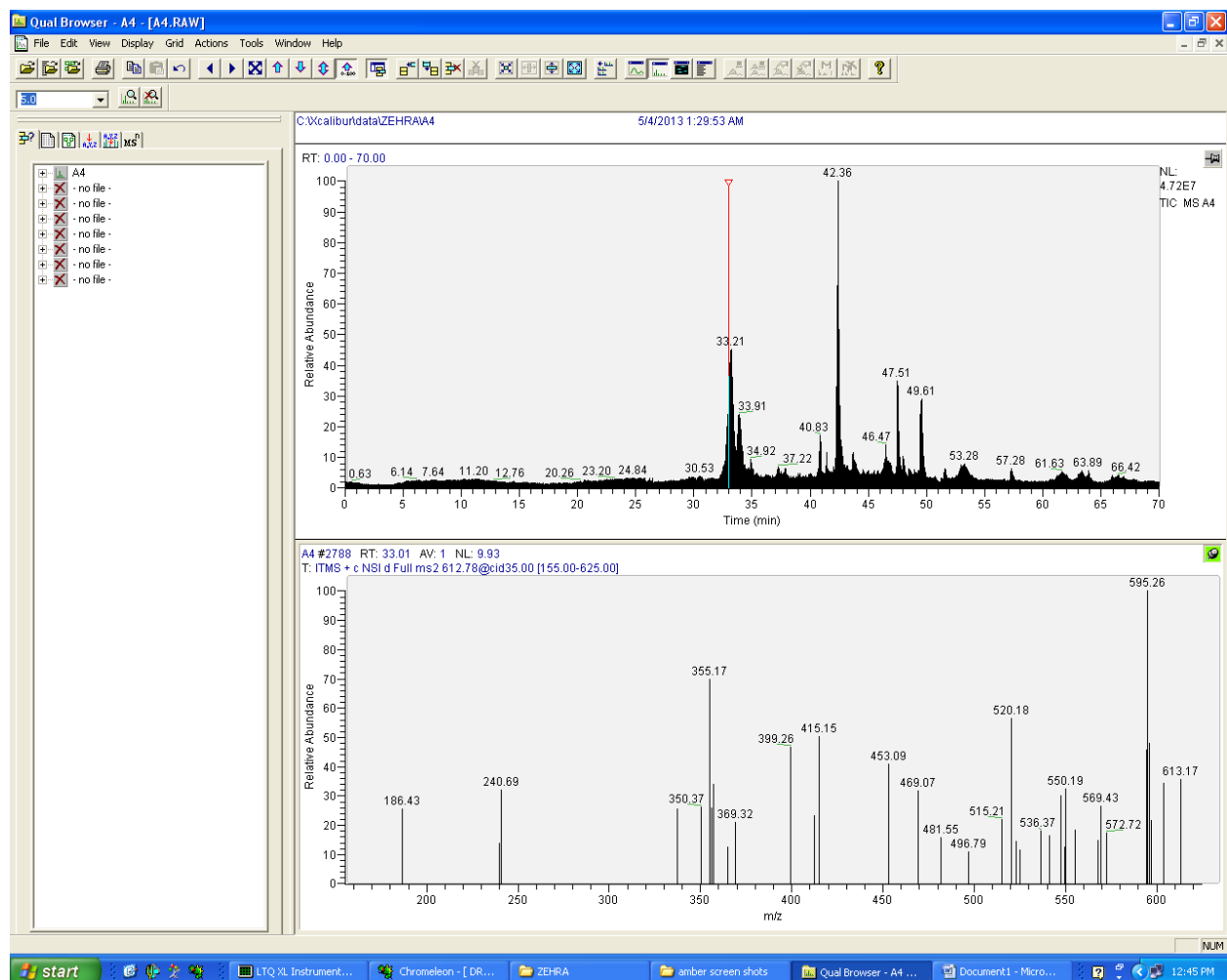

Supplement: Supplementary file 1 — Screen shots of RAW files of all identified proteins generated by Thermo LTQ XL linear trap Mass spectrometer. These MS/MS aggregated scans show the relative abundance of peptides in particular protein sample. The RAW files are converted into MGF files using Proteome Discoverer 2.0 software. [file 532953.f1.pdf]
